# Short- and long-term consequences of developmental saline stress: impacts on anuran respiration and behaviour

**DOI:** 10.1098/rsos.150640

**Published:** 2016-02-24

**Authors:** Brian D. Kearney, Phillip G. Byrne, Richard D. Reina

**Affiliations:** 1 School of Biological Sciences, Monash University, Clayton, Victoria 3800, Australia; 2 The Institute for Conservation Biology and Environmental Management, School of Biological Sciences, University of Wollongong, Wollongong, New South Wales 2522, Australia

**Keywords:** anthropogenic disturbance, foraging behaviour, escape response, *Litoria ewingii*, *Limnodynastes peronii*, metabolism

## Abstract

Secondary salinization has been identified as a major stressor to amphibians. Exposure to elevated salinity necessitates physiological adjustments and biochemical changes that may be energetically demanding. As such, exposure to non-lethal levels of salinity during development could potentially alter anuran metabolic rates and individual performance in both pre- and post-metamorphic life stages. We investigated the effects of non-lethal levels of salinity on metamorphic traits (time to reach metamorphosis and metamorphic mass), tadpole oxygen consumption, escape response behaviour (pre- and post-metamorphosis) and foraging ability post-metamorphosis in two native Australian frog species, the southern brown tree frog (*Litoria ewingii*) and the striped marsh frog (*Limnodynastes peronii*). We found that both *Lit. ewingii* and *Lim. peronii* exhibited differences in metamorphic traits in response to elevated salinity. Neither species showed significant change in oxygen consumption during development in response to salinity, relative to freshwater controls. Both species displayed impaired escape response behaviours in response to salinity during larval development, but flow-on effects to adult escape response behaviours and foraging performance were species-specific. Our results show that the influence of stressors during development can have consequences for anuran physiology and behaviour at multiple life stages, and emphasize the need for studies that examine the energetics of anuran responses in order to better understand the responses of biota to stressful environments.

## 1. Background

Amphibian numbers are in decline worldwide [1–3]. Members of this class are particularly sensitive to a broad range of threatening processes, including habitat destruction, climate change, disease, introduction of non-native species and environmental contamination [2,4]. Of particular concern has been the salinization of amphibian habitats via secondary salinization, chemical runoff and saltwater intrusion associated with sea-level rise [5–7]. Owing to the increased frequency of anthropogenic salinization in freshwater systems, there is a need for studies that directly examine the consequences of elevated salinity on amphibians in order to better manage declining populations [8,9].

A number of studies have examined the effect of increased salinity on individual anuran populations and it is now generally accepted that, while anuran susceptibility to salinity varies broadly across species [9], an increase in environmental salinity will impact anuran survival by interfering with key processes such as osmoregulation and growth [10,11]. However, there is a need for studies that directly examine the changes that can occur when anurans encounter environmentally relevant concentrations of salinity (i.e. elevated salinity levels likely to be encountered in their natural environment due to anthropogenic influences) that fall below the lethal limit, particularly during development [12,13], which, while insufficient to impact on survival directly, can lead to physiological, ecological and behavioural changes [14–16]. In addition, amphibians breed in brackish water more commonly than originally thought [15,17,18], so understanding these sub-lethal salinity effects during development on anuran biology and ecology is of increasing relevance.

In anurans, exposure to salt necessitates physiological adjustments and biochemical changes that are often energetically demanding [19]. For example, Wu *et al.* [20] determined that *Fejervarya limnocharis* exposed to elevated salinity increase production of Na^+^, K^+^-ATPase enzymes required for active transport of ions in osmotically stressful environments. These adjustments are thought to come at the cost of reallocating or diverting energy away from activities such as swimming and feeding, in order to meet increased osmoregulatory demands [15]. Anuran larvae have been shown to alter their metabolism in response to changed energy demands, such as long-term exposure to predators [21], UV exposure [22] and coal ash pollution [23]. Whole-animal oxygen consumption rate is commonly used as a proxy measure of total aerobic metabolic rate (e.g. [24]). If tadpoles increase oxygen consumption (and metabolic scope) in stressful environments in order to meet an increased energetic demand, they will reduce the need to trade-off energy from other activities. Therefore, in order to fully understand the potential costs of energetic trade-offs of tadpole development in stressful environments, there is a need for studies that examine these trade-offs in conjunction with any potential changes in oxygen consumption.

Studies of various anuran species have shown that exposure to elevated salinity typically retards tadpole growth and ultimately reduces animal body size [10,14,15,17]. A smaller body size is in turn known to reduce post-metamorphic survival [17], delay sexual maturity [25] and weaken cellular immune function in adults [26]. Furthermore, there is emerging evidence that such effects on individuals can reduce recruitment and negatively impact the viability of breeding populations [27–29]. However, given that the increased costs of osmoregulation in high saline environments could potentially reduce energy allocation to physical performance [15], there is a need for studies that examine the effects of saline exposure on animal performance beyond the effects on morphology and developmental rates during metamorphic stages.

Throughout all motile stages of the amphibian life cycle, there are strong selective pressures for effective escape behaviours when animals are faced with potentially threatening stimuli [30], and these behaviours are often modified in a changing environment [31]. Before metamorphosis, aquatic contaminants even at sub-lethal concentrations have been shown to induce fitness-altering changes in anuran behaviour [32–34]. For example, Squires *et al*. [35] demonstrated that tadpoles of *Litoria ewingii* in elevated salinity were less active and less able to avoid predation than those in lower salinity treatments. In addition, the truncation or acceleration of development that can occur in changing environments incurs a number of costs during the transition between the larval and adult environments [36]. Locomotor performance is a crucial measure in determining the survival and early growth of newly metamorphosed anurans [37], and studies have shown that jumping performance of metamorphs can be altered by environmental conditions during development [31,37,38]. This is a critical period for survival because individuals entering a novel habitat can be highly vulnerable to predators or the uncertainty of finding food [31]. As such, any attempts to manage anuran populations in salinized regions will need to consider the effects of salinity on animal locomotor ability both before and after metamorphosis.

We investigated the effects of non-lethal levels of salinity on oxygen consumption and behaviour in two native Australian frog species, the southern brown tree frog, *Litoria ewingii*, and the striped marsh frog, *Limnodynastes peronii*, throughout larval development and into the post-metamorphic life stage. Specifically, we examined the effects of salinity on: (i) oxygen consumption throughout tadpole development, (ii) tadpole escape response behaviour throughout development, (iii) frog escape response behaviour at metamorphosis and after metamorphosis, and (iv) foraging behaviour post-metamorphosis. In particular, we aimed to determine whether the effects of salinity exposure could be examined independently from the associated effects of body size both during and after the larval developmental phase. We hypothesized that animals reared in salinity would either increase oxygen consumption in order to meet the increased energetic demands associated with osmoregulation, or need to trade-off energy allocated for development and animal performance, thereby potentially reducing body size, delaying metamorphosis or reducing locomotor ability. In addition, we predicted that any potential alterations to developmental rates and morphology due to elevated salinity exposure would negatively impact on animal performance post-metamorphosis.

Anuran responses to salinity have been shown to differ between species [9,39]; therefore, any attempts to model the effects of environmental salinity on anuran species will need to consider that responses may differ between species. Both *Lit. ewingii* and *Lim. peronii* are common anuran species in southeastern Australia and both have been widely used in laboratory based studies of physiology and behaviour [34,40,41]. *Litoria ewingii*’s responses and tolerance to salinity have been well studied (e.g. [6,9,35,39]), but little data exist on the response of *Lim. peronii* to salinity [6]. Both species inhabit regions currently under threat from anthropogenic salinization [6], but they differ in both developmental period and post-metamorphic morphology [42]. Understanding both the long- and short-term effects of stressors is vital for managing freshwater habitats undergoing environmental change.

## 2. Material and methods

### 2.1 Experimental design

*Litoria ewingii* egg clutches (*n*=8 clutches; approx. 1–2 days post-oviposition) were obtained from sites in Beaufort (37°26′ S, 143°23′ E) and Clayton (37°55′ S, 145°07′ E), Victoria, Australia. *Limnodynastes peronii* egg clutches (*n*=11 clutches; approx. 1–3 days post-oviposition) were collected from San Remo (38°32′ S, 145°23′ E) and Silvan (37°49′ S, 145°25′ E), Victoria, Australia. Salinities at the sites ranged from 0.1 to 0.4 mS cm^−1^ (less than 0.28 ppt). Upon hatching, tadpoles from each site were haphazardly separated into one of two salinity treatments: 4.2 ppt (12% seawater) and 0.14 ppt (0.4% seawater). A salinity level of 4.2 ppt was determined to be high enough to cause developmental stress, while still falling below the lethal limit for early stage larvae of these species [6,9]. Tadpoles were housed in groups of 15–20 in 10 l (25×20×20 cm) containers holding 5 l of treatment water (*Lit. ewingii* *n*=10 containers per treatment, *Lim. peronii* *n*=8 containers per treatment). Tadpoles were housed collectively in order to more accurately reflect environmental tadpole densities and the effects associated with intra-specific competition [43], and because previous studies have indicated no interaction between salinity and tadpole density on tadpole development [44]. Tadpoles from these source treatments were haphazardly selected for each individual respiration, escape response and foraging response test and animals were not re-used for multiple tests nor repeated tests in the same experiments.

Tadpole oxygen consumption (mg O_2_ consumed per tadpole gram per hour; *Lit. ewingii:* *n*=13, *Lim. peronii:* *n*=13) and tadpole escape response (burst speed; *Lit. ewingii:* *n*=20, *Lim. peronii:* *n*=20) were recorded for both species at developmental stages 26–28 and stages 40–42 (Gosner). These stages were selected as they are easily distinguishable by non-intrusive visual observation. Frog locomotor ability (jump distance; *Lit. ewingii:* *n*=13, *Lim. peronii:* *n*=13) was tested at the completion of metamorphosis and again at 21 days post-metamorphosis, as during this period experimental animals had transitioned to an exclusively terrestrial life stage. Frog prey capture ability (time to prey capture and attempts at prey capture [45]) was recorded at 21 days post-metamorphosis (*Lit. ewingii:* *n*=18, *Lim. peronii:* *n*=17).

### 2.2 Metamorphic traits

Time to reach metamorphosis and mass at metamorphosis were measured for each individual. Mass of experimental animals was also recorded before all performance trials. To measure mass, each animal was removed from the treatment container, blotted dry using paper towel and weighed to the nearest 0.01 g (Shimadzu balance, Kyoto, Japan).

### 2.3 Tadpole oxygen consumption

Tadpole oxygen consumption rates were recorded by measuring oxygen depletion from within sealed water-filled respiratory chambers [22,24]. Larvae were starved for 48 h before measurement to reach a post-absorptive state. The influence of natural circadian rhythms on tadpole respiration was standardized by starting each trial at approximately the same time each day.

For each trial, tadpoles were blotted dry and weighed to the nearest 0.01 g before being placed in a custom made sealed chamber completely filled with treatment water (either 4.22 ppt NaCl equivalent or 0.14 ppt depending on treatment, at 20°C). The chamber consisted of a single 100 ml cylindrical plastic container (≈50 × 50 mm) with a 10 mm hole in the top into which an oxymeter (Wp-82, TPS, Brendale, Australia; ±0.2%; salinity corrected) was inserted and sealed with silicone grease. Water movement was maintained in the chamber via a magnetic stirring bar on a slow rotation. Tadpoles were given 20 min to get accustomed to the chamber, after which chamber oxygen concentration was measured. Chamber oxygen concentration was measured again 4–6 h later. The period of 4–6 h was chosen to ensure that animals were never in hypoxic water (oxygen content <3 mg O_2_ l^−1^ [46]). Standard controls filled with treatment water, but no tadpole, were used to account for microorganism respiration in the water and probe oxygen consumption, and instruments were calibrated before each successive trial. Tadpole oxygen consumption (mg O_2_ h^−1^) was calculated from the change in oxygen content relative to control, and standardized against chamber volume (ml) and duration in the chamber (h).

### 2.4 Tadpole escape response behaviour (swimming performance)

Tadpoles were placed into PVC testing channel (1000 × 30 × 30 mm), marked at 1 mm intervals and filled to 2 cm depth with treatment water. After a 5 min rest period, we videorecorded (Geovision GB-800; Irvine USA; 120 fps) the tadpoles as they were gently poked at the base of the tail with a pipette [35]. The burst speed (flight response) from the initial poke to the time the tadpole stopped swimming was calculated from the recordings using the video timer and marked lines on the test arena as reference (e.g. [47]). Tadpoles were stimulated five times with a 1 min rest period in between each recording. The fastest time for each tadpole was standardized against tadpole length (body lengths s^−1^) and used as the performance measure.

### 2.5 Adult escape response behaviour (jumping performance)

Before each trial, frogs were weighed to the nearest 0.01 g, and mass was used as a covariate for analysis to account for mass related differences in performance. Frogs were then placed in the centre of a rectangular PVC arena measuring 85 × 60 cm. Frogs were rested for a period of 5 min in the arena, after which time they were stimulated to jump with a gentle prod with a pipette at the tip of the vent. This is a common method for measuring frog performance (e.g. [41]). Each frog was stimulated to jump five times with a 1 min rest period in between each stimulus. The longest jump (initial vent position to final vent position) for each frog was recorded and used as the hopping distance and performance measure.

### 2.6 Adult foraging performance

Frogs were fed two crickets (Gryllacridinae spp., 3–6 days old, 2.5–4 mm long) and then starved for 48 h before each trial. After the starvation period, frogs were weighed to the nearest 0.01 g, and then placed in the centre of a rectangular arena of 85 × 60 cm. Frogs were rested for 5 min to allow for them to get accustomed to the arena and observers, after which a cricket was placed approximately 30 cm directly in front of the frog’s position, facing the frog. Observations ended after the frog successfully captured the cricket, or after 15 min, whichever came first. Only trials when the frog captured the cricket were used, with time (s) to prey capture used as the measure of foraging performance.

### 2.7 Husbandry techniques

For all experimental treatments, laboratory grade filtered water (Millipore) was mixed with Ocean Nature Synthetic Sea Salt (Aquasonic; Wauchope, Australia) to achieve the desired salinity level. Sea salt was used to make salt solutions as sodium chloride does not provide the necessary ions for proper osmoregulation [48], and because it more closely reflects natural salts found in inland Australia [49]. To prevent osmotic shock, salinity changes were performed gradually via 50% water change every 24 h over 3 days to achieve the desired treatment concentration.

Water temperatures were maintained at 20°C (±2°C) and water levels and salinity were monitored twice weekly and adjusted as necessary to maintain consistent salt concentrations. Partial (≈70%) water changes were made once per week in order to prevent water fouling. Metamorphs were housed individually in shaded 1 l containers with a moist terrestrial substrate (sphagnum moss). Treatment containers were arranged randomly on benches in a laboratory. In the laboratory, animals experienced natural light–dark cycles throughout development and were fed *ad libitum* on a mixture of frozen endive and *Spirulina* algal flakes (Nutrafin Max; West Yorkshire, UK). When not being fasted for performance assays, metamorphs were fed twice a week on a mixed diet of live crickets (Gryllacridinae spp.) and fruit flies (*Drosophila melanogaster*).

### 2.8 Statistical analysis

One-way ANOVAs were used to determine differences between salinity treatment group time to metamorphosis (days) and mass at metamorphosis (g). ANCOVAs were used to determine differences in respiration rates (mg O_2_ h^−1^) between salinity treatments, with tadpole mass (g) used as a covariate. In the case of burst speed, two-way ANOVAs were used with treatment and tadpole developmental stage as factors, and burst speed as the response variable. In post-metamorphic performance assays, because there is generally a significant relationship between animal size and performance [37], treatment effects were examined using ANCOVAs with individual mass as a covariate. We checked the parametric assumptions of the models for each treatment within each population, which necessitated log-transforming the ‘time to metamorphosis’ data before analysis. All analyses were conducted using StatView v. 5.0.1. (SAS Institution Inc., North Carolina, USA).

## 3. Results

### 3.1 Effect of salinity on metamorphic traits

Salinity exposure did not have a significant effect on the time to reach metamorphosis in either species (table 1), but did have a significant effect on body size in both species, with animals reared in 4.2 ppt being on average about 27% smaller than those reared in 0.14 ppt (table 1). However, differences in mass were no longer apparent three weeks post-metamorphosis (table 1).

**Table 1.** Metamorphic traits, oxygen consumption, escape response and foraging response behaviours of *Lit. ewingii* and *Lim. peronii* reared in two salinity treatments (0.14 ppt and 4.2 ppt.) Results shown as mean ± 1 s.e.m. Bold text denotes significant *p*-values (ANOVAs).

	*Litoria ewingii*				*Limnodynastes peronii*			
	0.14 ppt	4.20 ppt	*F*	*p*-value	0.14 ppt	4.20 ppt	*F*	*p*-value
metamorphic traits								
time to metamorphosis (days)	140.5 ± 4.1	135.2 ± 6.6	1.04	0.31	322.6 ± 6.1	339.6 ± 11.5	1.94	0.17
mass at metamorphosis (g)	0.30 ± 0.01	0.22 ± 0.01	34.96	**<0.0001**	1.03 ± 0.04	0.76 ± 0.05	17.94	**<0.001**
mass at three weeks post-metamorphosis (g)	0.33 ± 0.02	0.30 ± 0.02	1.29	0.27	1.21 ± 0.05	1.14 ± 0.05	4.91	0.5
tadpole oxygen consumption								
oxygen consumption at stage 26–28 (mg O_2_ g^−1^ h^−1^)	0.232 ± 0.010	0.223 ± 0.006	0.33^a^	0.57^a^	0.184 ± 0.009	0.186 ± 0.006	4.05^a^	0.57^a^
mass at stage 26–28 (g)	0.29 ± 0.03	0.20 ± 0.01	7.05	**0.01**	0.36 ± 0.03	0.30 ± 0.03	2.15	0.16
oxygen consumption at stage 40–42 (mg O_2_ g^−1^ h^−1^)	0.228 ± 0.002	0.232 ± 0.005	0.02^a^	0.88^a^	0.171 ± 0.006	0.180 ± 0.003	1.55^a^	0.23^a^
mass at stage 26–28 (g)	0.62 ± 0.03	0.50 ± 0.04	6.02	**0.02**	1.71 ± 0.06	1.34 ± 0.06	21.54	**<0.001**
tadpole escape response								
burst speed at stage 26–28 (body length s^−1^)	2.88 ± 0.13	2.22 ± 0.14	12.57	**0.001**	4.30 ± 0.11	2.69 ± 0.19	51.8	**<0.001**
burst speed at stage 40–42 (body length s^−1^)	2.19 ± 0.06	1.85 ± 0.11	7.62	**<0.01**	1.64 ± 0.05	1.44 ± 0.05	7.15	**0.011**
tadpole stage × salinity interaction			2.13	0.15			35.01	**<0.01**
adult locomotive ability								
jump distance at metamorphosis (cm)	12.69 ± 0.64	10.36 ± 0.75	5.54^a^	**0.03**^a^	15.35 ± 1.08	14.09 ± 0.89	0.82^a^	0.37^a^
jump distance at 3 weeks post-metamorphosis (cm)	13.92 ± 0.78	13.80 ± 0.72	0.01^a^	0.91^a^	14.82 ± 0.81	15.12 ± 0.87	0.08^a^	0.78^a^
adult feeding ability								
time to prey capture (s)	65.56 ± 7.25	45.89 ± 3.5	6.07^a^	**0.02**^a^	70.82 ± 5.19	65.62 ± 7.25	2.29^a^	0.14^a^

^a^Results of ANCOVA with animal mass as covariate.

### 3.2 Effect of salinity treatment on tadpole oxygen consumption

Tadpole oxygen consumption was significantly related to animal mass in both species (ANCOVA; *Lit. ewingii:* *F*=2227, *p*<0.001; *Lim. peronii*: *F*=1325, *p*<0.001; table 1); however neither *Lit. ewingii* nor *Lim. peronii* showed significant differences in oxygen consumption between salinity treatments at either of the tested developmental stages (table 1). There was no significant interaction between animal mass and animal oxygen consumption in either species (ANCOVA; *Lit. ewingii:* *F*=1.25, *p*=0.25; *Lim. peronii:* *F*=0.336, *p*=0.56).

### 3.3 Effect of salinity treatment on escape performance

In both species, tadpoles exposed to salinity had a significantly slower burst speed per body length at both stages of development (table 1). In the case of *Lim. peronii*, there was a significant interaction between development stage and salinity, with early stage tadpoles in high salinity having a greater reduction in burst speed then those at high salinity at a later stage in development (table 1). There was no interaction effect between development stage and salinity in *Lit. ewingii*.

While there was a significant effect of body size on adult jumping performance in both species, when controlling for the effects of body size, newly metamorphosed *Lit. ewingii* reared in 4.2 ppt had a significantly shorter jump distance than individuals reared in 0.14 ppt (table 1). Three weeks after metamorphosis the differences in jump distance were no longer significant (table 1). There was no significant effect of salinity treatment on escape performance at either stage in *Lim. peronii* (table 1).

### 3.4 Effect of salinity treatment on foraging performance

In *Lit. ewingii,* individuals raised in 4.2 ppt were significantly faster at capturing prey then those reared in 0.14 ppt (table 1). In *Lim. peronii,* there was no significant difference between treatment groups in time to capture prey.

## 4. Discussion

The aim of our study was to investigate the effects of non-lethal salinity levels on the growth, development and post-metamorphic performance of two frog species: *Lit. ewingii* and *Lim. peronii.* We found that tadpoles of both *Lit. ewingii* and *Lim. peronii* exhibited differences in metamorphic traits in response to salinity, resulting in animals reared in 4.2 ppt reaching metamorphosis in a comparable time, but at lower body size than those reared in 0.14 ppt. A similar developmental response to elevated salinity has been recorded in several of anuran species, including *Bufo calamita* and *Fejervarya limnocharis* [10,17]. Gomez-Mestre *et al*. [15] hypothesized that retarded growth and smaller body size at metamorphosis could be the outcome of energy normally allocated to growth being diverted into salt excretion and osmo- and iono-regulations, reducing the maximum body size attained during the developmental phase [50,51]. Such trade-offs are expected to be costly to post-metamorphic reproductive success and fitness, as a smaller mass at metamorphosis has been shown to reduce post-metamorphic survival [17], delay sexual maturity [25] and ultimately reduce recruitment and the viability of breeding populations [27–29].

Mass-specific oxygen consumption rates were not different between salinity treatment groups in either *Lit. ewingii* or *Lim. peronii.* A central component of energy budgeting theories is that an organism’s assimilation of energy forms a reserve that is then divided among growth, maintenance, maturation and reproduction [52]. In situations where a developing anuran has an increased somatic maintenance energy requirement, the tadpole will need to either increase its energy assimilation (and metabolic rate) or divert energy away from growth, maturation and/or other maintenance costs to meet the increased energy demands [50]. Our results show either that elevated salinity does not increase the energy demands of tadpoles, or that any increase in energy being allocated to maintain homeostatic balance is diverted from other energy requirements such as growth or performance. However, later stage tadpoles are tolerant to higher concentrations of salts than those tested here [13]; therefore, it is possible that in more stressful salinity concentration oxygen consumption rates may change. Therefore, future studies will need to explicitly examine the energy density and ATPase activity in developing tadpoles in response to elevated salinity.

In both *Lit. ewingii* and *Lim. peronii*, tadpoles exposed to salinity had a significantly lower burst speed. Our results support those of Squires *et al*. [35] and Gomez-Mestre *et al.* [15] in that additional osmoregulation not only diverts energy from somatic growth, but also reduces the energy available for physical performance. Alternatively, it may be that exposure to salinity could have a direct effect on tadpole muscle performance [45]. The reduction in body size and burst speed is expected to be costly, particularly in the early stage of development, as early stage tadpoles suffered a greater proportional reduction in burst speed than later stage tadpoles in *Lim. peronii*. Not only is burst speed an important anti-predator response in tadpoles (e.g. [53]), but also many predators of anuran larvae are gape-limited and have a preference for smaller prey [54]. For example, Arendt [55] demonstrated that slower and smaller tadpole morphs of the New Mexico spadefoot toad (*Spea* *multiplicata*) were more likely to be eaten by predatory tadpoles of the same species. A number of studies have shown a physiological trade-off between growth rate and burst speed (e.g. [56,57]), but our study demonstrates that this effect can be independent of tadpole size and may be a result of reduced energy allocated to physical performance in stressful environments.

Increased hopping distance has previously been associated with improved likelihood of survival [58]. Variation in anuran jumping performance is usually attributed to body size or condition, with larger individuals generally showing greater short-term performance and endurance performance [59], due to either longer legs acting as extended levers during jumping [60] or an increased musculature or energy storage in muscle tissues [61]. Although there was a correlation between body mass and hoping distance in both our study species, after controlling for variation in individual body size, we still found evidence for a reduction in jumping performance in *Lit. ewingii* reared in 4.2 ppt compared to those reared in 0.14 ppt. However, differences in jumping performance were no longer significant three weeks after metamorphosis. At three weeks post-metamorphosis, frogs are likely to be more reliant on energy assimilated from prey sources than on lipid stores [62]. It is possible that the reliance on external energy sourced from prey items during this later stage impacted upon locomotive ability and that some compensatory improvement in performance of salinity-affected animals resulted.

In this study, *Lit. ewingii* raised in 4.2 ppt were significantly faster to capture prey than those reared in 0.14 ppt. In addition, at three weeks post-metamorphosis, there were detectable differences in animal mass between treatments in either species. This result may indicate that while *Lit. ewingii* reared in elevated salinity are at a disadvantage in terms of both mass-reduced body size and locomotor ability, they invest more energy in prey capture in order to more quickly regain mass as a form of compensatory growth. There also exists the potential for over-compensation after exposure to stress during development. For example, Hector *et al*. [41] found that *Lit. ewingii* tadpoles that compensated for dietary restrictions early in life were larger and performed better in both foraging and locomotion trials in later life compared with individuals from control groups. Based on these findings, it is likely that post-metamorphic responses to developmental stress vary in response to both type and magnitude of the stressor.

In contrast to our findings for *Lit. ewingii*, there was no effect of salinity treatment on hopping performance or prey capture ability foraging performance in *Lim. peronii*, indicating that post-metamorphic effects of salinity exposure are likely to be species-specific. In agreement with previous studies’ descriptions of species metamorphic traits (reported in [63]), *Lim. peronii* took over twice as long to reach metamorphosis and were over three times larger than *Lit. ewingii.* This may indicate that any differences in anuran performance due to exposure to a stressor during development are dependent on the species absolute metamorphic traits (i.e. the behaviour of larger or slower to develop anuran species is less impacted by exposure to salinity than smaller or faster developing anuran species). Nevertheless, if *Lim. peronii* anurans can maintain locomotive and prey capture ability despite the costs associated with development in high saline environments, this will provide site-specific advantages, as the increased salt concentrations could decrease competition and predation from less salt-tolerant species. However, there are likely to be other costs of altered development in a stressful environment. For example, Gervasi & Foufopoulos [26] found that although tadpoles exposed to desiccation had similar metamorphic mass and body morphology to normally hydrated animals, they had weaker cellular immune function post-metamorphosis. Our results emphasize that the developmental responses of anurans to environmental stress are highly variable and that further research into the impact of salinity on amphibian fitness in later life stages is required across a diversity of species.

In conclusion, we investigated the effects of elevated salinity on fitness-determining traits in anurans before and after metamorphosis. We found that both *Lit. ewingii* and *Lim. peronii* exhibited differences in metamorphic traits in response to elevated salinity, resulting in animals reaching metamorphosis in a comparable time, but at lower body size than animals reared in lower salinity treatments. In both species, tadpoles displayed reduced burst speed during development, but no changes in overall oxygen consumption. Furthermore, flow-on effects to adult hopping performance and foraging performance were both species- and age-specific. Empirical studies testing the responses of freshwater animals to salinity fluctuations are urgently needed, because there remains a limited understanding of the energetic and life-history trade-offs that individuals face when developing in stressful environments. This knowledge has the potential to aid in the management of biodiversity in habitats affected by anthropogenic salinization.

## Supplementary Material

Experimental data for short and long-term consequences of developmental saline stress: Impacts on anuran respiration and behaviour

## Data accessibility

See the electronic supplementary material to obtain data used in this paper.
